# 1800. *Candida Auris and Neighborhood Socioeconomic Vulnerability in Maryland*

**DOI:** 10.1093/ofid/ofad500.1629

**Published:** 2023-11-27

**Authors:** Lauren Leigh Smith, Jason Falvey, David Blythe, Elisabeth Vaeth, Jamie Rubin, Rebecca Perlmutter, Clare Rock, Mary-Claire Roghmann, Surbhi Leekha

**Affiliations:** University of Maryland, Baltimore, MD; University of Maryland, Baltimore, MD; Maryland Department of Health, Baltimore, Maryland; Maryland Department of Health, Baltimore, Maryland, Baltimore, Maryland; MDH, Baltimore, Maryland; Maryland Department of Health, Baltimore, Maryland; Johns Hopkins School of Medicine, Baltimore, Maryland; University of Maryland School of Medicine, Baltimore, MD; University of Maryland School of Medicine, Baltimore, MD

## Abstract

**Background:**

*Candida auris* is an emerging fungal pathogen increasingly recognized as a cause of healthcare-associated infections including outbreaks. The first case of transmission was detected in Maryland in June 2019. This study aims to describe characteristics of patients with C. auris and determine if patients were more likely to reside in a disadvantaged neighborhood or receive care from a nursing home in a disadvantaged area.

**Figure 1.**
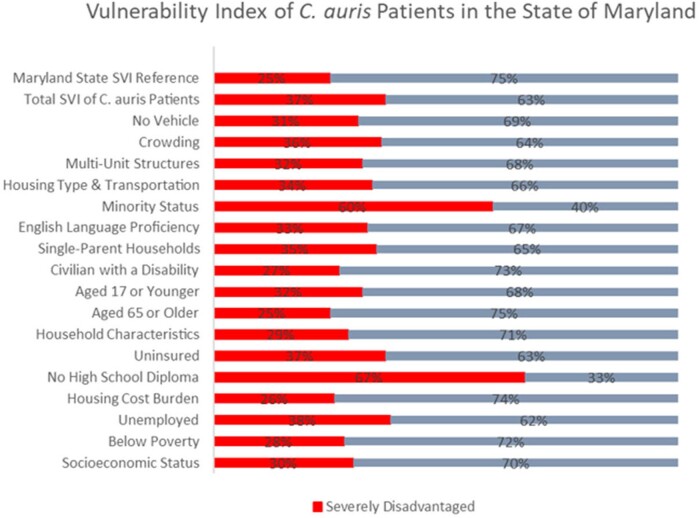
Vulnerability Index of C. auris Patients in the State of Maryland. Severely disadvantaged is defined as SVI percentile ranking greater than or equal to the 75th percentile. Red indicates the percentage of C. auris patients living in severely disadvantaged census tracts.

**Methods:**

We performed a descriptive analysis of all cases of C. auris among Maryland residents from June 2019 to December 2021. Maryland Department of Health conducted active surveillance through point prevalence surveys around newly detected cases. We geocoded patient home addresses and skilled nursing facilities (SNFs) with outbreaks to assign a census block group or tract. The social vulnerability index (SVI) and the area deprivation index (ADI) were obtained at the state level, with an SVI ≥ 75th percentile or an ADI ≥ 80th percentile considered severely disadvantaged consistent with prior work.

**Results:**

140 individuals tested positive for *C. auris* in the study period. The median age was 68 (IQR 53-74), and 91 (65%) case-patients were male.  46 (33%) of case-patients had a positive clinical culture while 100 were detected on surveillance PCR-positive screening swabs.  60 (43%) of case-patients resided at a SNF.  37 (26%) of case-patients were ventilated and 87 (62%) had a documented wound.

33 (28%) case-patients, with an identifiable address, resided in severely disadvantaged neighborhoods.  *C. auris* patients disproportionately came from neighborhoods with higher crowding, higher proportions of uninsured, and racial/ethnic minority status (Figure 1). 30% of SNFs with *C. auris* transmission were in neighborhoods in the bottom 20th percentile of state neighborhood socioeconomic disadvantage.

**Conclusion:**

Neighborhood socioeconomic vulnerability may play a role in the emergence and transmission of C. auris in a community. Further work is needed to understand patient-level risk factors as well as how staffing levels and care environments in SNFs are affected by neighborhood socioeconomic factors.

**Disclosures:**

**Lauren Leigh Smith, MD, MAS**, LEAP Fellowship: Grant/Research Support

